# Shikonin Inhibits Tumor Growth of ESCC by suppressing PKM2 mediated Aerobic Glycolysis and STAT3 Phosphorylation

**DOI:** 10.7150/jca.58494

**Published:** 2021-06-11

**Authors:** Qiqi Zhang, Qing Liu, Shutao Zheng, Tao Liu, Lifei Yang, Xiujuan Han, Xiaomei Lu

**Affiliations:** 1 State Key Laboratory of Pathogenesis, Prevention and Treatment of High Incidence Diseases in Central Asia, Clinical Medical Research Institute, The First Affiliated Hospital of Xinjiang Medical University, Urumqi, Xinjiang Uygur Autonomous Region, China.; 2 Department of Clinical Laboratory, First Affiliated Hospital of Xinjiang Medical University, Urumqi, Xinjiang Uygur Autonomous Region, China.; 3 Cancer Hospital Affiliated of Xinjiang Medical University, Urumqi, Xinjiang Uygur Autonomous Region, China.

**Keywords:** Shikonin, Pyruvate kinase 2 (PKM2), Aerobic glycolysis, Esophageal squamous cell carcinoma (ESCC)

## Abstract

**Background:** Shikonin, a small molecule inhibitor of pyruvate kinase 2 (PKM2), has been demonstrated to play the antitumor effect in various cancers. However, the specific effects and related regulatory mechanism of Shikonin in esophageal squamous cell carcinoma (ESCC) have not been clearly declared.

**Materials and methods:** Cell viability was valued through 3-(4,5-Dimethylthiazol-2-yl)-2,5-diphenyltetrazolium bromide (MTT) assay. Glucose consumption, lactate production, glycolytic intermediates and pyruvate kinase enzymatic activity were measured using corresponding assay kits. Patient-derived xenograft (PDX) models were constructed to observe the anti-ESCC effect of Shikonin *in vivo*. PKM2, p-PKM2, signal transducer and activator of transcription 3 (STAT3), p-STAT3, glucose transporter 1 (GLUT1) and hexokinase 2 (HK2) in ESCC tissues were assessed by western blot. The expression of PKM2, p-PKM2, p-STAT3, GLUT1 and HK2 was assessed by immunohistochemistry (IHC) in ESCC tissue based on PDXs.

**Results:** Shikonin effectively inhibited cell proliferation in dose-dependent and time-dependent manner compared with the control group. The detection of glycolysis showed that Shikonin suppressed the glucose consumption, lactate production, glycolytic intermediates and pyruvate kinase enzymatic activity. Furthermore, Shikonin not only inhibited the growth of ESCC, but also decreased the expression of p-PKM2 and p-STAT3 *in vivo*. Finally, Shikonin suppressed the expression of GLUT1 and HK2 proteins which are related to glycolysis.

**Conclusion:** Shikonin has a significant antitumor effect in the ESCC by suppressing PKM2 mediated aerobic glycolysis and regulating PKM2/STAT3 signal pathway.

## Introduction

Esophageal cancer (EC) is the seventh most prevalent malignancy and sixth highest cause of cancer‐related mortality worldwide 1. Esophageal squamous cell carcinoma (ESCC) is the most common subtype of EC in China and the 5-year mortality rate is up to 70% 2. Thus, it is urgent to find new drugs to treat ESCC.

Aerobic glycolysis is one of the characteristics of tumor cells. The metabolic characteristics of aerobic glycolysis are known as the Warburg effect, characterized by high glucose uptake rate, high lactate production 3. The conversion of phosphoenolpyruvate to pyruvate is the last step in glycolysis, which is catalyzed by pyruvate kinase (PK) 4. The pyruvate kinase M2 (PKM2) is a rate-limiting enzyme in the last step of glycolysis of tumor cells, and plays a key role in glycolysis 5. Measurement of PKM2 in serum samples was performed using ELISA in our group's previous study. Average serum PKM2 level in eight normal individuals was 13.55 ng/mL, and average serum PKM2 level in 35 Kazakh's ESCC patients was 78.84 ng/mL, nearly five times higher than that in normal individuals 6. In addition, the expression of PKM2 was detected in 139 paraffin-embedded human ESCC and paired NAT samples and the results showed that PKM2 was highly expressed in ESCC tissues and high PKM2 expression was significantly correlated with metastasis and poor clinical prognosis 7. Therefore, reagents that inhibit aerobic glycolysis, especially those that regulate PKM2 activity, have shown great potential in the development of antitumor drugs 8.

Shikonin, an active component extracted from Radix Arnebiae, has been reported to possess anti-microbial, anti-inflammatory properties in various cells 9, 10. It has also been demonstrated to play the antitumor effect in various cancers. A recent study suggested that Shikonin inhibited growth, invasion and glycolysis of nasopharyngeal carcinoma cells through inactivating the phosphatidylinositol 3 kinase/AKT signal pathway 11. Liu et al proved that Shikonin inhibited proliferation and glycolysis and induced cell apoptosis by inhibiting PKM2 in hepatocellular carcinoma cells 12. James et al believed that Shikonin, as an inhibitor of PKM2, reduced the proliferation, migration and induced cell death of pancreatic ductal adenocarcinoma cells due to its inhibition of glycolysis, ATP depletion, plasma membrane calcium pump and cytotoxic Ca overload 13. However, it is not clear whether Shikonin can be used as an effective reagent against ESCC *in vitro* and *in vivo*.

Patient-derived xenograft (PDX) models have been developed for translating basic research studies to clinical applications 14. By directly transplanting the fresh tumor tissue of patients into the immunodeficient mice, the model can maintain the biological characteristics of the primary tumor, the genetic characteristics similar to the patients and the tumor heterogeneity, and play an irreplaceable role in the precision treatment research of clinical tumor. Currently, PDX models have been used in many kinds of tumor research, especially in the study of tumor drug resistance 15-20.

In present study, the effect of Shikonin on ESCC *in vitro* and *in vivo* was investigated. Our results show that Shikonin has a significant antitumor effect in the ESCC by suppressing PKM2 mediated aerobic glycolysis and regulating PKM2/STAT3 signal pathway.

## Materials and methods

### GEPIA (gene expression profiling interactive analysis) and GeneCards (The Human Gene Database)

The mRNA expression of pyruvate kinase M (PKM) was assessed by analyzing a dataset downloaded from GEPIA (http://gepia.cancer-pku.cn/index.html). The GEPIA online database contains research data both from TCGA and GTEx, including RNA sequencing data of 9736 tumor samples and 857 normal samples.

The protein expression of PKM was assessed by analyzing a dataset downloaded from GeneCards (https://www.genecards.org/). The GeneCards is a searchable, integrative database that provides comprehensive, user-friendly information on all annotated and predicted human genes. The knowledgebase automatically integrates gene-centric data from ~150 web sources, including genomic, transcriptomic, proteomic, genetic, clinical and functional information.

### Cell culture

Human ESCC cell lines, KYSE150 was obtained from the Shanghai Institute of Cell Biology, Chinese Academy of Sciences (Shanghai, China), Eca109 was obtained from Wuhan University (Wuhan, China). All cells were maintained in Roswell Park Memorial Institute (RPMI) 1640 medium (Gibco, Carlsbad, CA) supplemented with 10% fetal bovine serum (FBS; Gibco) and 1% penicillin-streptomycin within a humidified atmosphere containing 5%CO_2_ at 37 °C. For evaluating the effect of Shikonin on the viability and metabolic status of cells, different concentration of Shikonin (MCE, Shanghai, China) were added into cell culture medium and cells were incubated for 72 h.

### Lentiviral transfection

Lentiviruses carrying green fluorescent protein (GFP) along with scrambled (Lv-shRNA-control and Lv-overexpress-control), PKM2-overexpressing construct (Lv-overexpress-PKM2), and PKM2 shRNA (Lv-shRNA-PKM2) were purchased from Shanghai Gene Pharma Co Ltd (Shanghai, China). For infection, KYSE150 and Eca109 cells were incubated with lentiviruses using polybrene (5 μg/mL) and enhanced infection solution according to the manufacturer's protocol. After 72 h, all fluorescent cells were screened via flow cytometry and transfection efficiency evaluated via western blot.

### Cell proliferation

Cell proliferation was assayed by MTT (3-(4,5-Dimethylthiazol-2-yl)-2,5-diphenyltetrazolium bromide) assay using MTT Cell Proliferation and Cytotoxicity Assay Kit (Solarbio, Beijing, China) according to the manufacturer's protocol. Briefly, Eca109 cells were seeded onto 96-well plates (Corning) at a density of 1×10^4^ cells per well in RPMI 1640 medium and incubated for 24 h (37 °C and 5%CO_2_). The medium was then replaced with either serum-free RPMI 1640 medium or serum-free RPMI 1640 medium containing various concentrations (0, 2, 5, 10, 20 or 50 µM) of Shikonin (the total volume in each well was 200 µL). After incubation for another 24 h, 48 h, and 72 h, the number of viable cells was determined by measurement of the absorbance (OD 490 nm).

### Analysis of glucose metabolism

Cells were seeded into 25 cm^2^ culture flasks, and after 24 h the culture medium was replaced with fresh complete medium and incubated for additional 48 h. The medium was then replaced with either serum-free RPMI 1640 medium or serum-free RPMI 1640 medium containing 20 µM of Shikonin. After incubation for another 24 h, the media were then collected for measurement of glucose, and lactate concentration and cells harvested for protein lysates. In addition, 1×10^6^ cells were collected for the measurement of intracellular lactate concentration. Glucose levels were determined using a glucose assay kit (Tongwei, Shanghai, China). Glucose consumption was calculated by deducting the measured glucose concentration in the media from the original glucose concentration. Lactate levels were determined using a lactate assay kit (Tongwei, Shanghai, China) according to the manufacturer's instruction. Considering the cell number of each sample may be different, all the levels of glucose or production of lactate were finally normalized to the protein level.

### Quantification of glycolytic intermediates and pyruvate kinase enzymatic activity

2,3-Disphosphoglycerate (2,3-DPG) levels were determined using a 2,3-DPG assay kit (TSZ, USA) and values were normalized on the basis of the protein assay. 1×10^6^ cells were collected for the measurement of intracellular Glucose 6-phosphate (G6P), ATP, NADPH and PK activity. G6P levels were determined using a G6P assay kit (Beyotime, Shanghai, China). ATP levels were determined using an ATP assay kit (Beyotime, Shanghai, China). NADPH levels were determined using the NADP^+^/NADPH quantification kit (Beyotime, Shanghai, China). The levels of PK activity were determined using the Pyruvate Kinase Activity Colorimetric/Fluorometric Assay Kit (Biovision). All testing process performed according to the manufacturer's protocol.

### PDX animal experiment

BALB/c nude female mice (Beijing Anikeeper Biotech Co., Ltd, Beijing, China) were used for animal experiment when the mice were 6 to 8 weeks old and weighed 17.1 g to 20.9 g. All mice were fed in SPF grade. This study was approved by the Ethics Committee of the First Affiliated Hospital of Xinjiang Medical University (Urumqi, Xinjiang Uygur Autonomous Region, China). Two cases of ESCC were constructed in PDX animal experiment and designated as ES0195 and ES0172. BALB/c nude mice were subcutaneously inoculated with ES0195 and ES0172 tumor masses of 2-3 mm to establish ESCC tumor models. When tumors reached an average volume of about 150 mm^3^, mice were randomly divided into two groups according to tumor size for further experiment as follows: (I) vehicle-treated group (n=5); (II) group treated with 2 mg/kg of Shikonin (n=5). Mice in both groups were intraperitoneally injected once a day for 7 days. Tumor volume was calculated from measurements of 2 diameters of the individual tumor base using the following formula: tumor volume (mm^3^) = (length×width^2^) × 0.5. Tumor growth was routinely monitored and the effects of drug administration on the normal behavior of animals were routinely monitored, including activity of experimental animals, food intake and water intake, weight gain or loss (weight was measured twice a week) and other abnormal conditions. Mice were monitored until 28 days, at which time mice were euthanized and tumors extracted. Shikonin (catalog number: HY-N0822/CS-5906) was respectively obtained from MCE.

### Western blot analysis

Tumor samples were grouped and lysed with radioimmunoprecipitation assay (RIPA) buffer (catalog number:899001; Thermo) containing HALT protease and phosphatase inhibitor (catalog number: 78442; Thermo), 0.5M EDTA (catalog number: 20-158; Millipore), 0.5M EGTA (catalog number: ST-068; Beyotime), glycerol (catalog number: G5516; Sigma), complete mini protease inhibitor (EDTA-free) (catalog number:11836170001; Roche), phosphatase inhibitor cocktail A+B (catalog number: B15001; Bimake) and phenylmethanesulfonyl fluoride (PMSF) Solution (100 mM) (catalog number:93482-50ML-F; Sigma). The concentration of all the proteins was determined by a BCA kit (Solarbio, Beijing, China). The proteins were separated by running a 10% SDS-PAGE gel and were then transblotted to PVDF membranes. Then, the membranes were blocked with 5% skim milk for 2 h and probed with the following primary antibodies overnight at 4 °C and with secondary antibodies (AP-conjugated goat anti-rabbit or anti-mouse) for 2 h. Novex® AP Chromogenic substrate (BCIP/NBT) was used to visualize the protein bands. ImageJ software (1.8.0, USA) was used to semi-quantitatively analyze the protein bands. Primary antibodies were as follows: anti‐PKM2 (1:1000, 4053 S; Cell Signaling Technology, Beverly, MA), anti‐p-PKM2 (1:1000, 3827 S; Cell Signaling Technology, Beverly, MA), anti‐signal transducer and activator of transcription 3 (STAT3) (1:1000, 9139 S; Cell Signaling Technology, Beverly, MA), anti‐p-STAT3 (Y705, 1:2000, 9145 S; Cell Signaling Technology, Beverly, MA), anti‐glucose transporter 1 (GLUT1) (1:1000, 66290‐1‐Ig; Proteintech), anti‐hexokinase 2 (HK2) (1:2000, 22029‐1‐AP; Proteintech) and anti‐glyceraldehyde 3‐phosphate dehydrogenase (GAPDH) (1:10000, ab8245; Abcam).

### Immunohistochemical analysis

Tumor tissues embedded in paraffin were subjected to hematoxylin and eosin (H&E) staining and immunohistochemistry (IHC). Tissue samples were dewaxed and rehydrated, submerged into EDTA antigenic retrieval buffer, and treated with 3% hydrogen peroxide. Slides were incubated with the following primary antibodies overnight at 4 °C and with secondary antibodies (Enzyme-conjugated goat anti-rabbit or anti-mouse) for 60 minutes at 37 °C. 3,30-Diaminobenzidine was used to visualize the target proteins and then counterstained with hematoxylin. After staining, the sections were photographed and analyzed using the ImageJ software (1.8.0, USA). Primary antibodies were as follows: anti‐PKM2 (1:800, 4053S; Cell Signaling Technology, Beverly, MA), anti‐p-PKM2 (1:400, 3827S; Cell Signaling Technology, Beverly, MA), anti‐p-STAT3 (Y705, 1:200, 9145S; Cell Signaling Technology, Beverly, MA), anti‐GLUT1 (1:400, 66290‐1‐Ig; Proteintech) and anti-HK2 (1:300, 22029‐1‐AP; Proteintech).

### Statistical analysis

All experiments were performed independently at least three times, unless otherwise stated. Statistical analyses were performed using SPSS 17.0 software (SPSS, Chicago, IL). GraphPad Prism 8.0 software (GraphPad Software, Inc., La Jolla, CA, USA) was used to draw the plots. The data are expressed as the mean ± standard deviation (SD). A two-tailed Student's t-test or One-way analysis of variance was used to evaluate the differences among groups. The LSD test was used for Analysis of variance post hoc testing.

## Results

### PKM2 promotes the aerobic glycolysis of ESCC

**Figure 1:**
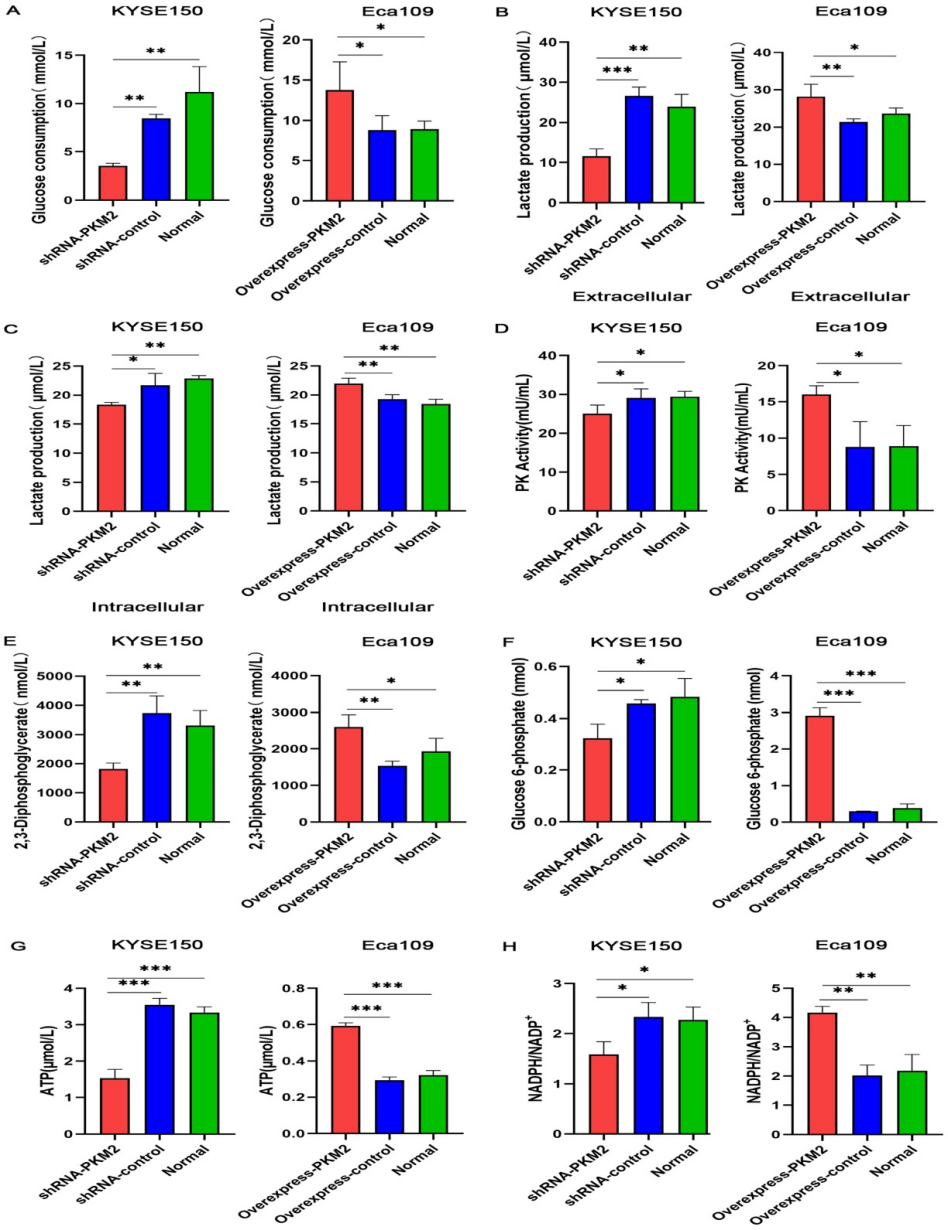
** PKM2 promotes the aerobic glycolysis of ESCC.** The levels of (A) glucose consumption, (B) extracellular lactate production, (C) intracellular lactate production, (D) PK activity, (E) 2,3-Diphosphoglycerate (2,3-DPG), (F) glucose 6-phosphate (G6P), (G) ATP, (H) and NADPH/NADP^+^ decreased in KYSE150 cells expressing PKM2 shRNA and increased in Eca109 cells overexpressing PKM2. Data shown are mean ± SD of n≥3 technical replicates and are representative of three independent experiments. P values were calculated by One-way analysis of variance. *P<0.05, **P<0.01, ***P<0.001.

To clarify the effect of PKM2 on aerobic glycolysis, the glucose consumption, lactate production, glycolytic intermediate and pyruvate kinase enzymatic activity after overexpression and silencing of PKM2 were detected by ELISA kits. Firstly, PKM mRNA expression profile across all tumor samples and paired normal tissues was obtained through GEPIA database (Figure S1A) and the protein expression in normal tissues and cell lines for PKM was obtained through GeneCards database (Figure S1B). According to the expression profiles, PKM gene was highly expressed in EC. In addition, our previous study 7 confirmed that PKM2 was highly expressed in ESCC tissues and PKM2 was highly expressed in KYSE150 cell line and low expressed in Eca109 cell line, so lentiviral transfection was used to silence PKM2 in KYSE150 cell line and overexpress PKM2 in Eca109 cell line. As shown in Figure 1 and Table S1, both glucose consumption and lactate production displayed elevation after PKM2 overexpression, as well as glycolytic intermediates and pyruvate kinase enzymatic activity significantly increased. On the contrary, glucose consumption, lactate production, glycolytic intermediates and pyruvate kinase enzymatic activity significantly decreased after PKM2 was silenced. The results suggest that the Warburg effect mediated by PKM2 can provide sufficient energy and a large number of glycolytic intermediates for the rapid proliferation of ESCC.

### Shikonin inhibits the proliferation of ESCC

**Figure 2:**
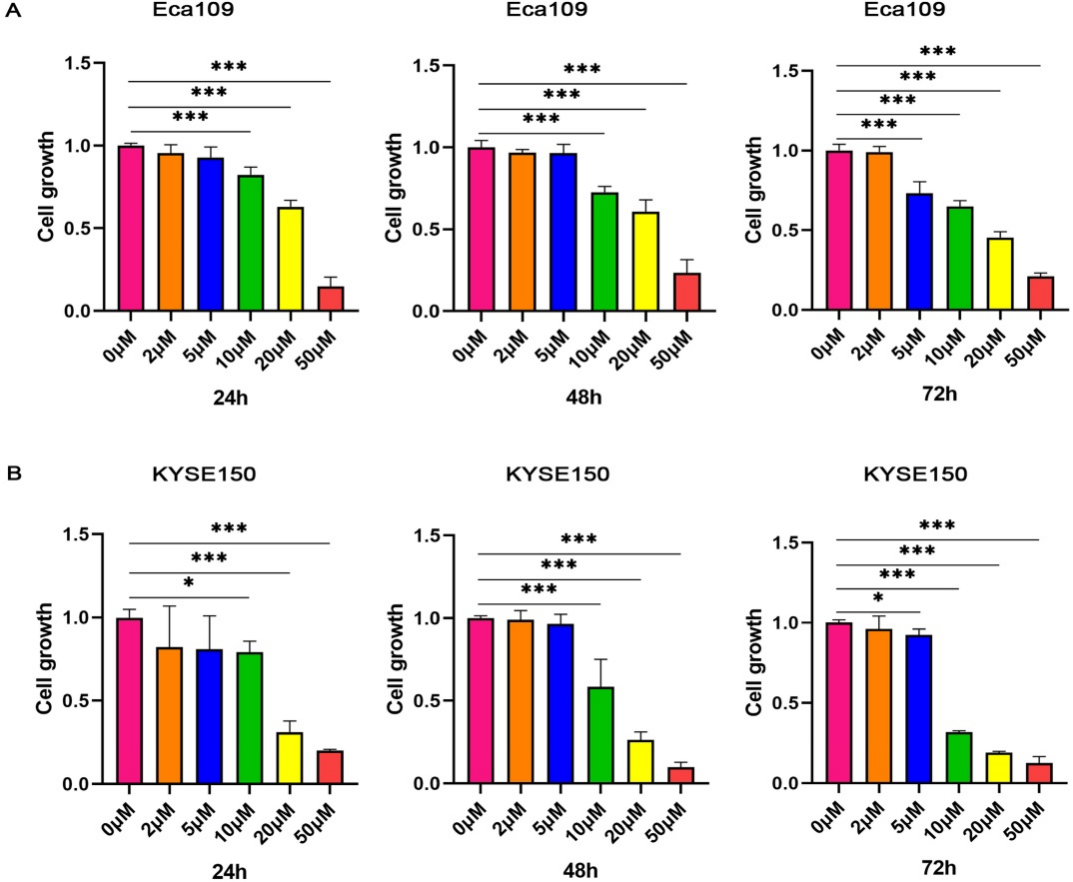
** Shikonin inhibits the proliferation of ESCC.** (A) Eca109 (B) and KYSE150 tumor cells were treated with various concentration (0, 2, 5, 10, 20 or 50 µM) of Shikonin for 72 h. Data shown are mean ± SD of n≥3 technical replicates and are representative of three independent experiments. P values were calculated by One‐way analysis of variance. *P<0.05, **P<0.01, ***P<0.001.

Shikonin, as an inhibitor of PKM2, has been reported to have antitumor effect. To test the effect of Shikonin on the growth of ESCC, the effects of different concentrations of Shikonin on the proliferation of KYSE150 and Eca109 tumor cells were determined. As shown in Figure 2 and Table S2, Shikonin inhibited the growth of KYSE150 and Eca109 tumor cells in a dose-dependent and time-dependent manner.

### Shikonin suppresses the aerobic glycolysis of ESCC

**Figure 3:**
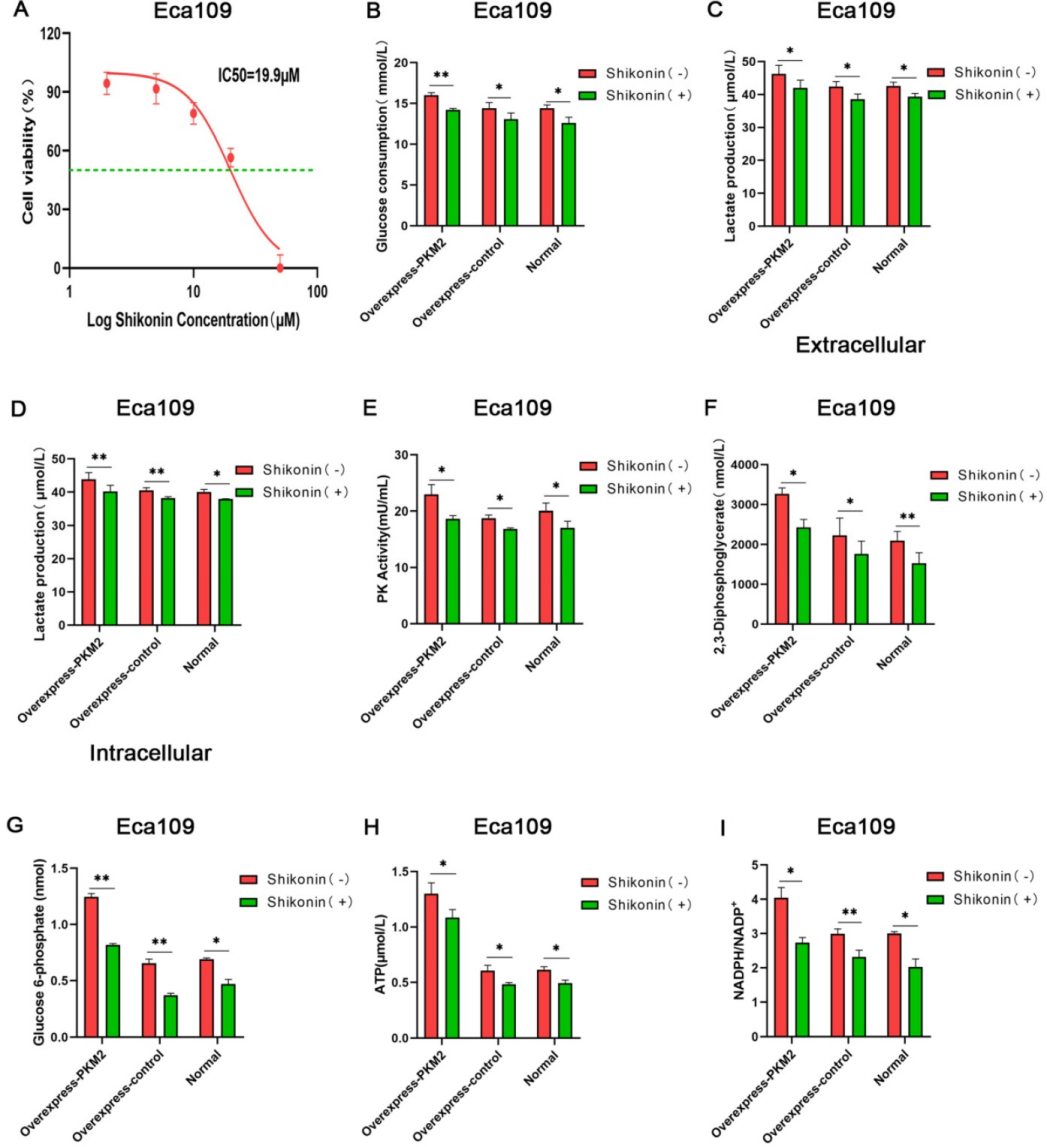
** Shikonin suppresses the aerobic glycolysis of ESCC.** (A) The half maximal inhibitory concentration (IC50) of Shikonin on Eca109 cells for 24 h. The levels of (B) glucose consumption, (C) extracellular lactate production, (D) intracellular lactate production, (E) PK activity, (F) 2,3-Diphosphoglycerate (2,3-DPG), (G) glucose 6-phosphate (G6P), (H) ATP, (I) and NADPH/NADP^+^ decreased in Eca109 cells overexpressing PKM2 after 24 h of Shikonin treatment at 20 µM concentration. Data shown are mean ± SD of n≥3 technical replicates and are representative of three independent experiments. P values were calculated by Paired-samples t-test. *P<0.05, **P<0.01, ***P<0.001.

Given that aerobic glycolysis is a key for most tumor cells to maintain rapid growth and metastasis and PKM2 is the key metabolic enzyme for glycolysis, whether Shikonin inhibits ESCC cell growth via blocking the aerobic glycolysis was tested. In present study, glucose consumption, lactate production, glycolytic intermediates, and pyruvate kinase enzymatic activity were measured in Eca109 cells with overexpressing PKM2, which were treated with Shikonin or not. According to the cell proliferation experiment, the half maximal inhibitory concentration (IC50) of Shikonin on Eca109 cells for 24 h was 19.9 µM (Figure 3A). Therefore, the relevant metabolic indexes of the Eca109 cells treated with 20 µM of Shikonin for 24 h were determined. The results showed that glucose consumption, lactate production, glycolytic intermediates and pyruvate kinase enzymatic activity significantly decreased after Shikonin treatment (Figure 3 and Table S3). It suggests that Shikonin suppresses the aerobic glycolysis of ESCC.

### Shikonin inhibits ESCC growth *in vivo*

**Figure 4:**
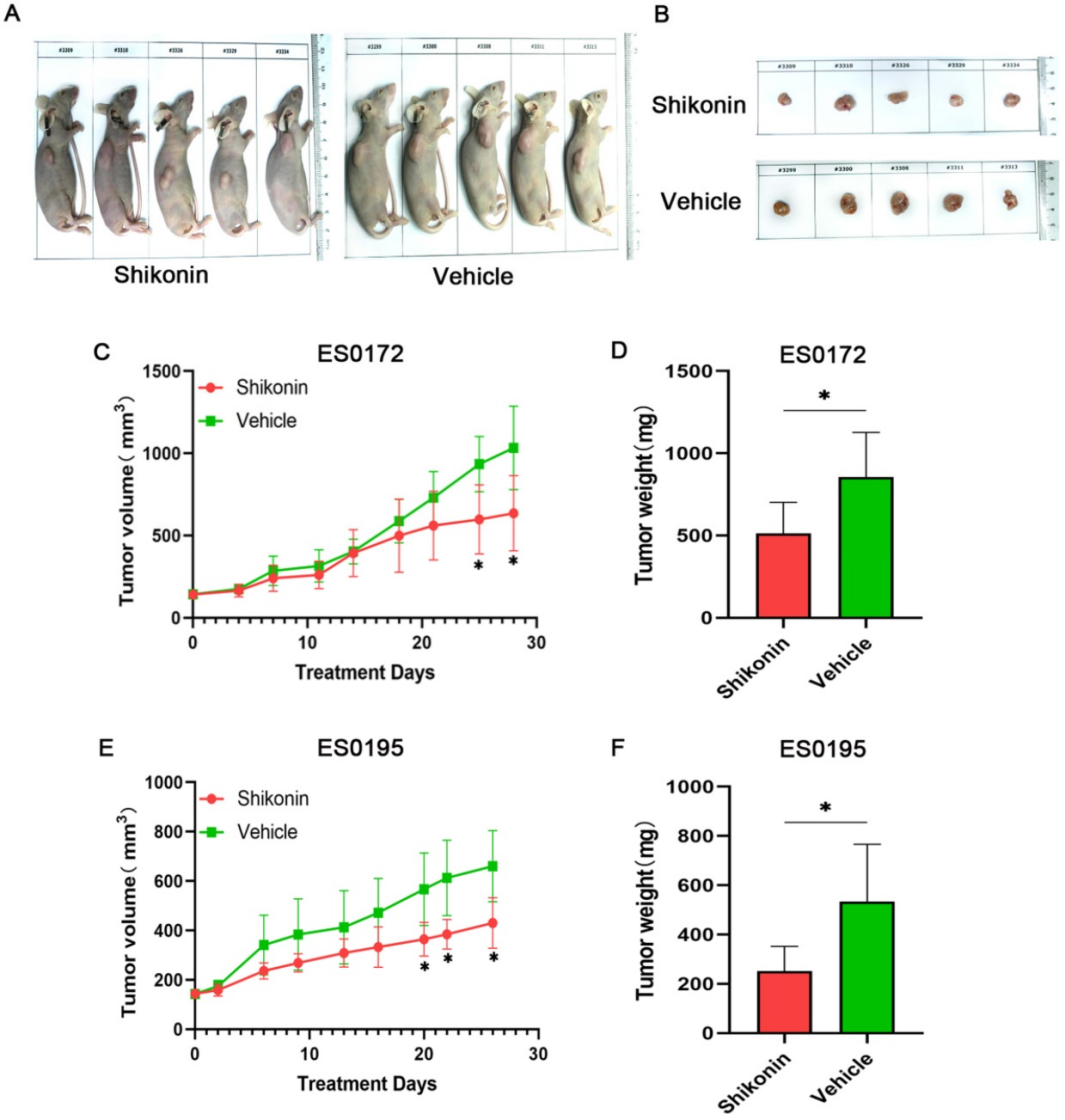
** Shikonin inhibits tumor growth *in vivo*. ES0172 and ES0195 PDX models were established to observe the antitumor effect of Shikonin *in vivo*.** Mice were treated with vehicle or Shikonin (2 mg/kg/day) for 7 days at the same time (n = 5 per group). (A, B) Representative images of the mice and tumor with subcutaneous xenograft ESCC. The xenograft ESCC in the mouse treated with Shikonin was significantly smaller than that in the control mouse. (C, E) The tumor growth curve. The results showed Shikonin inhibited the tumor growth (n = 5). (D, F) Weight of the vehicle control group and Shikonin treatment group. The results showed Shikonin inhibited the tumor growth (n = 5). Data are shown as mean ± SD. P values were calculated by Student's t-test. *P<0.05, **P<0.01, ***P<0.001.

In order to test the effect of Shikonin on ESCC growth *in vivo*, two patients were selected to establish ES0172 and ES0195 PDX models (Figure 4). In the two cases, Shikonin significantly inhibited the growth of ESCC compared with vehicle control group (Figure 4C, 4E, Table S4 and Table S5). In the ES0172 model, tumor weight in the Shikonin group decreased by approximately 40% compared with the vehicle control group, and in the ES0195 model, tumor weight in the Shikonin group decreased by approximately 53% compared with the vehicle control group (Figure 4D, 4F and Table S6).

### Shikonin inhibits tumor growth *in vivo* via regulating PKM2/STAT3 signal pathway and suppressing PKM2 mediated aerobic glycolysis

**Figure 5:**
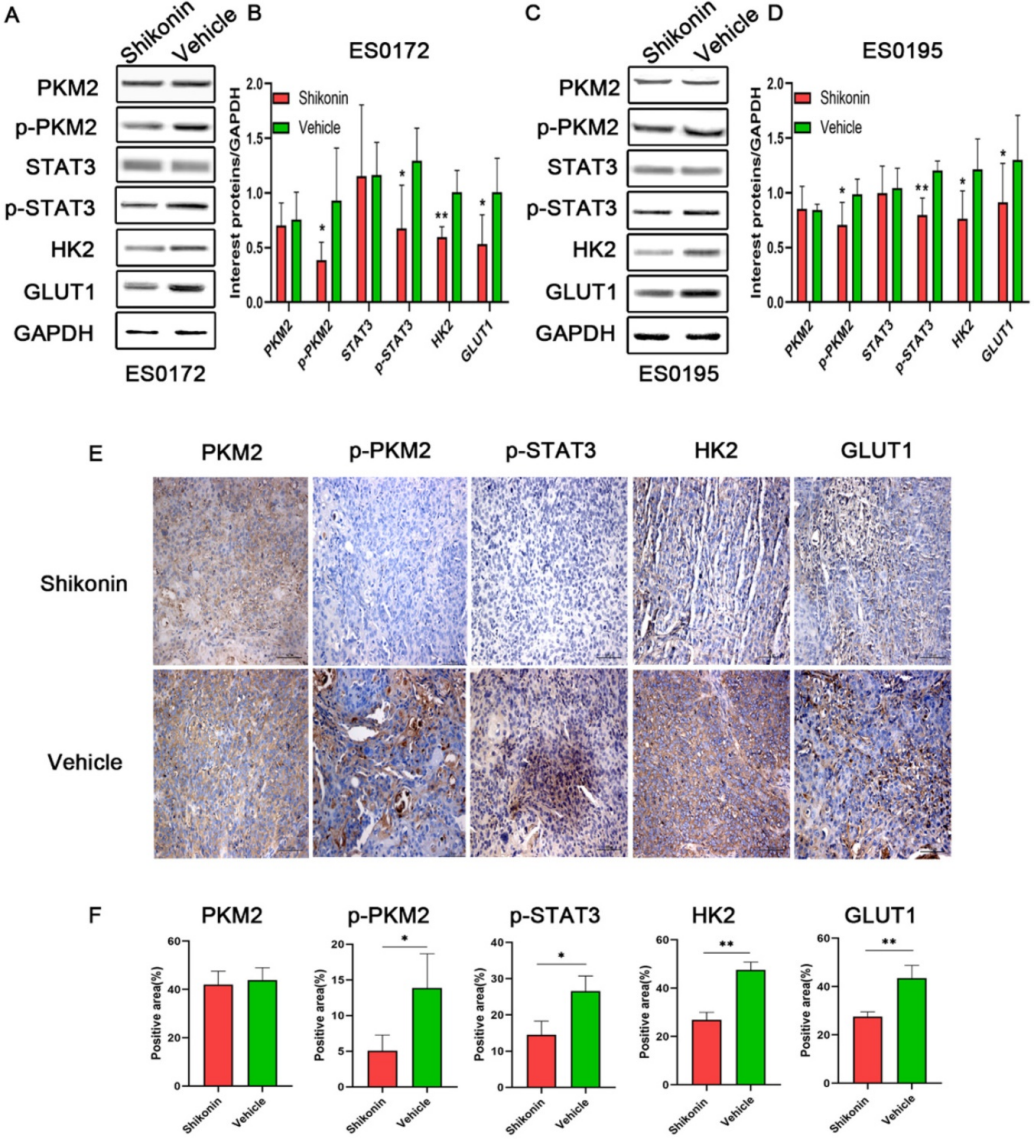
** Shikonin inhibits tumor growth *in vivo* via regulating PKM2/STAT3 signal pathway and suppressing PKM2 mediated aerobic glycolysis.** (A, C) Expression levels of PKM2, p-PKM2, STAT3, p‑STAT3, HK2 and GLUT1 proteins were detected by western blot in ES0172 and ES0195 PDX model tumors. (A, C) The qualitative (B, D) and quantitative results showed that the expression of p-PKM2, p-STAT3, HK2 and GLUT1 in Shikonin group was lower than that in vehicle control group. (E) Typical graphs of IHC staining of PKM2, p-PKM2, p‑STAT3, HK2 and GLUT1 in ES0172 and ES0195 PDX model tumor tissues (100 µm). (F) The quantitative results of IHC showed that the expression of p-PKM2, p-STAT3, HK2 and GLUT1 in Shikonin group was lower than that in vehicle control group. Data are shown as mean ± SD. P values were calculated by Student's t-test. *P<0.05, **P<0.01, ***P<0.001.

Further studies on ES0172 and ES0195 models were constructed in present study (Figure 5). In the two models, p-PKM2 expression in the Shikonin group was significantly lower than that in the vehicle control group, which was analyzed by western blot (Figure 5A-5D). Similarly, p-STAT3 expression was significantly different between the two groups. The expression of GLUT1 and HK2, which are related to glucose metabolism, was also detected. The results showed that the expression of GLUT1 and HK2 in the Shikonin group was significantly lower than those of the vehicle control group. The same results were confirmed by IHC (Figure 5E and 5F). These results suggest that Shikonin inhibits ESCC growth *in vivo* by regulating PKM2/STAT3 signal pathway and suppressing PKM2 mediated aerobic glycolysis.

## Discussion

Abnormal glucose metabolism is one of the basic characteristics of tumors, and PKM2 is a key speed-limiting enzyme in the Warburg effect. Therefore, in the present investigation, the inhibitory effect of Shikonin on ESCC *in vivo* and *in vitro* and its mechanism were studied. These results showed that Shikonin reduced ESCC burden *in vitro* and *in vivo*. The antitumor mechanisms of Shikonin may be associated with suppression of PKM2 mediated aerobic glycolysis and inhibition of PKM2/STAT3 signaling pathway in ESCC.

Many studies have reported that Shikonin has antitumor effect, which is mainly related to cycle, apoptosis, autophagy, necrosis and immune escape. The molecules and signaling pathways involved include Bcl-2, p53 21, cIAP1/2 22, P21 23, PD-L1 24, HIF1α/PKM2 25, PI3K/ AKT /mTOR 26, MAPK 27, NF-κB/STAT3 24 and so on. Compared with the previous reports, our study focused on the effect of Shikonin on glycolysis, which was consistent with the study of Zhao et al 8. In our study, glucose consumption and lactate production were not only measured, but glycolytic intermediates and pyruvate kinase enzymatic activity was also gauged in our study, which enriches the current understanding of Shikonin on PKM2-mediated aerobic glycolysis and non-mediated aerobic glycolysis. In addition, the PDX models were used to study the antitumor effect of Shikonin, which was another highlight deserving to be noted, making the results better translationally predictive of clinical efficacy. To our knowledge, it is the first time to investigate the antitumor effect of Shikonin and groping for its mechanism using the ESCC PDX models. Lastly, we also found that Shikonin inhibited the PKM2/STAT3 signaling pathway and inhibited the expression of GLUT1 and HK2, which has not been reported ever before. Taken as a whole, it is helpful to further understand the mechanism of Shikonin's inhibition of PKM2-mediated aerobic glycolysis and non-mediated aerobic glycolysis.

The oligomers of PKM2 exist in high activity tetramer and low activity dimer forms. Tetramers have pyruvate kinase activity and dimers have protein kinase activity. Under the regulation of various factors, PKM2 converts between tetramer and dimer, which not only accumulates a large number of intermediates in glucose metabolism, but also reprograms energy metabolism, and ultimately enables tumor cells to survive even in the absence of oxygen 28. In the present study, glucose consumption, lactate production, glycolytic intermediates, and pyruvate kinase enzymatic activity decreased after Shikonin treatment and ultimately failed to provide sufficient energy and metabolic intermediates for the growth of ESCC. However, we found that Shikonin inhibited p-PKM2 expression but not total PKM2. So, how does Shikonin block the aerobic glycolysis of ESCC by inhibiting p-PKM2? Yang et al 29 pointed out that the ERK pathway mediated PKM2 phosphorylation and then entered the nucleus, and nuclear PKM2 promoted the expression of glycolytic related proteins, thus promoting the glycolytic effect. Hexokinase (HK) is a rate-limiting enzyme that regulates the first step reaction of glycolytic pathway. Several studies 30-35 confirmed that HK2 expression is increased in various tumor tissues and is closely related to the progression of malignant tumors. GLUT1 is the primary transporter that facilitates glucose uptake 36, and plays a critical role in tumorigenesis and tumor progression in multiple cancer types 37. Therefore, the expression of GLUT1 and HK2 in ESCC after Shikonin treatment in the PDX models was detected in our study. Both western blot and IHC results indicated that expression of GLUT1 and HK2 decreased after Shikonin treatment. These results further suggest that Shikonin inhibits tumor growth by inhibiting PKM2 mediated aerobic glycolysis in ESCC. The mechanism was that Shikonin inhibited the expression of p-PKM2 and further reduced the expression of glycolysis related proteins. More researches are needed to clarify whether Shikonin directly inhibites pyruvate kinase activity. However, our data presented here are still informative.

STAT3 is a constitutively activated oncogenic protein in various human tumors and represents a valid target for anticancer drug design 38. The gene expression of many enzymes in glucose metabolism is regulated by the JAK/STAT3 pathway, and the role of PKM2/STAT3 pathway in cancer progression has attracted increasing attention. Therefore, the expression of p-STAT3 was detected. We found that Shikonin inhibited the growth of ESCC *in vivo* by down-regulating p-STAT3, which is regulated by p-PKM2.

In summary, Shikonin inhibited ESCC growth *in vitro* and *in vivo*. Furthermore, antitumor effect of Shikonin is to suppress PKM2 mediated aerobic glycolysis and to regulate PKM2/STAT3 signal pathway.

## Supplementary Material

Supplementary figure and tables.

## Abbreviations

EC: esophageal cancer
ESCC: esophageal squamous cell carcinoma
PK: pyruvate kinase
PKM2: pyruvate kinase M2
PDX: patient-derived xenograft
RPMI: roswell park memorial institute
FBS: fetal bovine serum
GFP: green fluorescent protein
2,3-DPG: 2,3-Disphosphoglycerate
G6P: glucose 6-phosphate
RIPA: radioimmunoprecipitation assay
STAT3: signal transducer and activator of transcription 3
GLUT1: glucose transporter 1
HK: hexokinase
HK2: hexokinase 2
GAPDH: glyceraldehyde 3‐phosphate dehydrogenase
IHC: immunohistochemistry
SD: standard deviation
SEM: standard error of the mean
