# Supplementary material for: Shikonin Inhibits Tumor Growth of ESCC by suppressing PKM2 mediated Aerobic Glycolysis and STAT3 Phosphorylation
Source: J Cancer. 2021 Jun 11;12(16):4830–40. doi: 10.7150/jca.58494 (PMC8247391; doi:10.7150/jca.58494)
Supplement: Supplementary file 1 — Supplementary figure and tables. [file jcav12p4830s1.pdf]

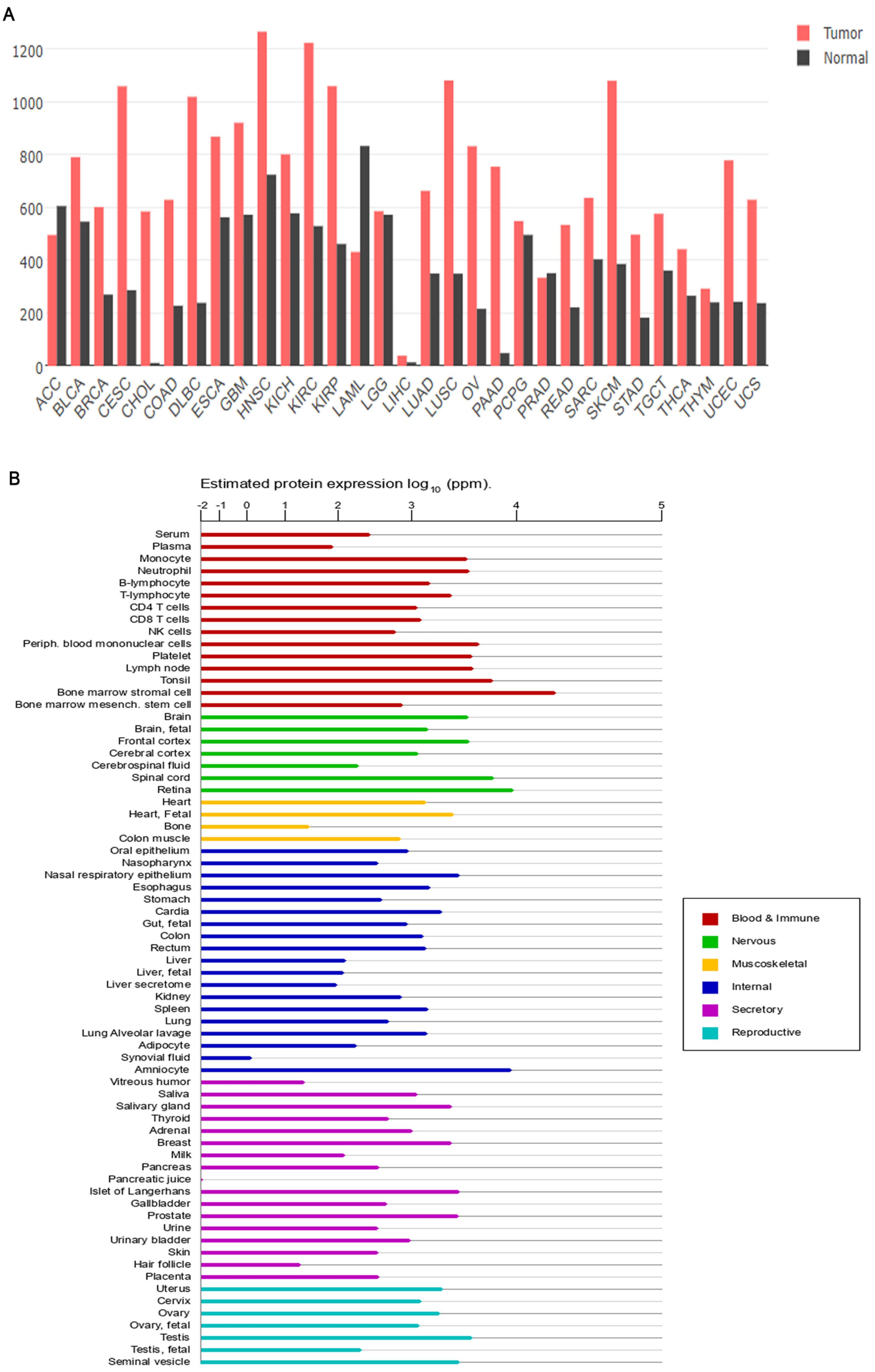

Figure S1. PKM expression profiles. (A) PKM mRNA expression profile across all tumor samples and paired normal tissues was obtained through GEPIA database. (B) The protein expression in normal tissues for PKM was obtained through GeneCards database.

Table S1. Values of glycolysis related indexes after PKM2 overexpression and silencing

Items (mean ± SD)	KYSE150			Eca109		
	shRNA	shRNA-control	Normal	Overexpress-PKM2	Overexpress-control	Normal
Glucose consumption (mmol/L)	3.54±0.26	8.47±0.43	11.21±2.62	13.75±3.50	8.79±1.80	8.93±0.10
Extracellular lactate production (μmol/L)	11.55±1.82	26.61±2.17	23.92±3.06	28.18±3.29	21.40±0.82	23.58±1.53
Intracellular lactate production (μmol/L)	18.34±0.38	21.72±2.01	22.89±0.48	21.97±0.91	19.26±0.80	18.41±0.81
PK Activity (mU/mL)	24.98±2.23	29.08±2.37	29.41±1.40	16.04±1.19	8.80±3.47	8.90±2.84
2,3-Diphosphoglycerate (nmol/L)	1816.70± 200.00	3738.90± 585.32	3316.70± 504.43	2605.60± 328.86	1538.90± 126.20	1927.80± 368.68
Glucose 6-phosphate (nmol)	0.32±0.06	0.46±0.02	0.48±0.07	2.92±0.21	0.29±0.01	0.38±0.12
ATP (μmol/L)	1.54±0.24	3.54±0.18	3.33±0.15	0.59±0.02	0.29±0.02	0.32±0.02
$NADPH/NADP^+$	1.59±0.25	2.33±0.29	2.27±0.26	4.16±0.21	2.02±0.35	2.18±0.55

Table S2. The effects of different concentrations of shikonin on the proliferation of ESCC cells

Shikonin	KYSE150 cell growth (mean±SD)			Eca109 cell growth (mean±SD)		
	24 h	48 h	72 h	24 h	48 h	72 h
0 μM	1.00±0.05	1.00±0.01	1.00±0.02	1.00±0.01	1.00±0.04	1.00±0.04
2 μM	0.82±0.25	0.99±0.06	0.96±0.08	0.96±0.05	0.97±0.02	0.99±0.04
5 μM	0.81±0.20	0.97±0.06	0.92±0.04	0.93±0.06	0.97±0.05	0.73±0.07
10 μM	0.79±0.07	0.59±0.17	0.32±0.01	0.82±0.05	0.73±0.04	0.65±0.04
20 μM	0.31±0.07	0.26±0.05	0.19±0.01	0.63±0.04	0.61±0.07	0.46±0.04
50 μM	0.20±0.01	0.10±0.03	0.13±0.04	0.15±0.06	0.23±0.08	0.21±0.02

Table S3. Values of glycolysis related indexes with shikonin treatment or not

Items (mean ± SD)	Overexpress-PKM2		Overexpress-control		Normal	
	Shikonin (−)	Shikonin (+)	Shikonin (−)	Shikonin (+)	Shikonin (−)	Shikonin (+)
Glucose consumption (mmol/L)	16.01±0.32	14.21±0.17	14.42±0.70	13.07±0.75	14.43±0.40	12.61±0.70
Extracellular lactate production (μmol/L)	46.27±2.60	41.98±2.38	42.40±1.52	38.59±1.54	42.61±1.12	39.30±1.03
Intracellular lactate production (μmol/L)	43.87±2.01	40.19±1.89	40.51±0.83	38.21±0.45	40.00±0.84	37.94±0.08
PK Activity (mU/mL)	22.96±1.75	18.68±0.55	18.79±0.55	16.88±0.19	20.09±1.33	17.09±1.13
2,3-Diphosphoglycerate (nmol/L)	3263.33± 152.75	2430.00± 200.00	2230.00± 435.89	1763.33± 321.46	2096.67± 230.94	1530.00± 264.58
Glucose 6-phosphate (nmol)	1.25±0.03	0.82±0.01	0.66±0.04	0.37±0.02	0.69±0.01	0.47±0.04
ATP (μmol/L)	1.30±0.10	1.08±0.07	0.61±0.05	0.48±0.02	0.61±0.03	0.49±0.03
$NADPH/NADP^+$	4.05±0.30	2.74±0.15	3.00±0.14	2.32±0.19	3.01±0.05	2.03±0.24

Table S4. Tumor volume in ES0172 model

Treatment Days	Shikonin (mean±SEM)	Vehicle (mean±SEM)
0	142.06±11.37	142.69±6.41
4	165.27±16.75	175.88±10.66
7	241.12±35.57	285.20±40.08
11	261.02±36.89	315.85±43.90
14	392.75±63.81	402.88±33.68
18	499.81±99.82	588.64±59.76
21	560.99±93.43	730.33±71.34
25	597.61±93.97	934.86±107.67
28	635.81±102.31	1032.68±113.66

Table S5. Tumor volume in ES0195 model

Treatment Days	Shikonin (mean±SEM)	Vehicle (mean±SEM)
0	143.82±7.71	143.11±4.69
2	158.76±10.68	174.47±10.46
6	236.20±14.46	340.93±54.19
9	268.45±16.67	383.79±64.45
13	308.80±25.43	412.74±66.49
16	332.54±36.59	472.33±62.07
20	364.83±30.65	566.68±65.59
22	384.41±26.82	613.28±68.39
26	430.37±45.95	660.43±64.63

Table S6. Tumor weight in ES0172 and ES0195 model

Model	Shikonin (mean±SEM)	Vehicle (mean±SEM)
ES0172	512.30±84.54	855.80±121.48
ES0195	251.76±44.93	533.74±103.82
